# MASH as an emerging cause of hepatocellular carcinoma: current knowledge and future perspectives

**DOI:** 10.1002/1878-0261.13685

**Published:** 2024-06-14

**Authors:** Michael Karin, Ju Youn Kim

**Affiliations:** ^1^ Laboratory of Gene Regulation and Signal Transduction, Departments of Pharmacology and Pathology, School of Medicine University of California San Diego La Jolla CA USA; ^2^ Department of Molecular and Life Science Hanyang University ERICA Ansan Korea

**Keywords:** ER stress, genetic mutation, Hepatocellular carcinoma, inflammation, lipotoxicity, MASH, metabolic syndrome, obesity

## Abstract

Hepatocellular carcinoma is one of the deadliest and fastest‐growing cancers. Among HCC etiologies, metabolic dysfunction‐associated fatty liver disease (MAFLD) has served as a major HCC driver due to its great potential for increasing cirrhosis. The obesogenic environment fosters a positive energy balance and results in a continuous rise of obesity and metabolic syndrome. However, it is difficult to understand how metabolic complications lead to the poor prognosis of liver diseases and which molecular mechanisms are underpinning MAFLD‐driven HCC development. Thus, suitable preclinical models that recapitulate human etiologies are essentially required. Numerous preclinical models have been created but not many mimicked anthropometric measures and the course of disease progression shown in the patients. Here we review the literature on adipose tissues, liver‐related HCC etiologies and recently discovered genetic mutation signatures found in MAFLD‐driven HCC patients. We also critically review current rodent models suggested for MAFLD‐driven HCC study.

## Graphical abstract

Metabolic dysfunction‐associated steatohepatitis is emerging as a leading cause of hepatocellular carcinoma in individuals whose lifestyle and dietary habits promote a positive energy balance. Here, we reviewed the role of free fatty acids in mediating lipotoxicity and its implications for organellar stress and genome alteration in hepatocytes. Additionally, we discussed the pathogenic processes underlying the malignant transformation of hepatocytes in obese patients.

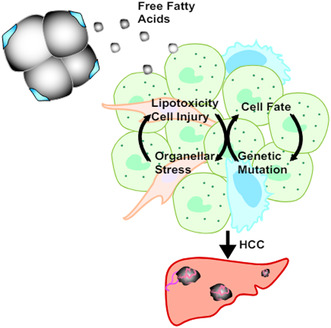

## 1 Introduction

Hepatocellular carcinoma (HCC) is a primary liver cancer, accounting for 84% of liver tumorigenesis, and is the 3rd leading cause of cancer‐related deaths [1]. HCC is a complex multifactorial disease. The personal characteristics, including age, gender, and race, vary in impact on HCC incidence, thereby making HCC extremely complex and associated with poor prognosis. After diagnosis, the median survival of HCC patients is approximately 6–20 months [2], indicating that HCC is a deadly liver cancer requiring intensive medical care. Although early detection allows better management of disease and increases the survival rate [3], HCC is typically diagnosed at the late stage, therefore interfering with many curable treatment options. According to the Surveillance, Epidemiology, and End Results (SEER) database, age‐adjusted HCC incidence was increased 2‐fold during the past two decades, with a corresponding raise in mortality and hospitalization rate [4]. This is mostly due to the increased incidence of cirrhosis and fibrosis caused by multiple HCC etiologies, including hepatitis B virus (HBV), hepatitis C virus (HCV), heavy alcohol drinking, and the obesity pandemic [5]. Moreover, HCC incidence began to occur in younger ages, implying that socio‐economic changes lead to early exposure to carcinogenic factors [6]. Thus, establishment of a surveillance program that detects HCC in its early stage is urgently required.

## 2 Etiology of HCC

**Fig. 1:**
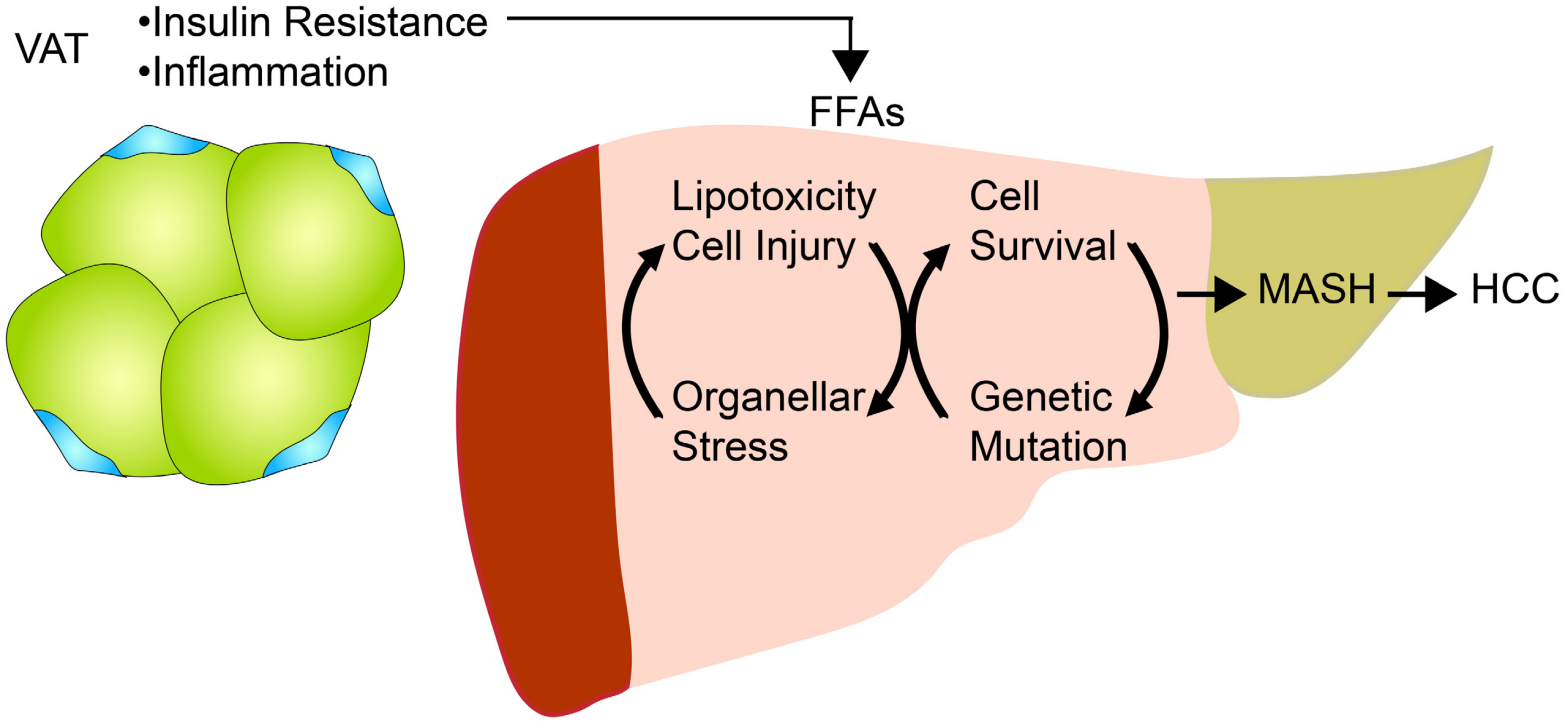
Pathophysiological course of MASH‐derived HCC along with the risk factors for disease progression. FFAs, Free fatty acids; HCC, Hepatocellular carcinoma; MASH, Metabolic dysfunction‐associated steatohepatitis; VAT, Visceral adipose tissue.

HCC is affected by multi‐etiological factors, all of which vary by geographic location, impact the characteristics of patients, and influence the course of disease progress [7]. Infection of hepatitis B virus (HBV) or hepatitis C virus (HCV) was one of leading causes of chronic liver diseases and implicated in onset of HCC. The incidence of HCC due to viral infections shows geographical preference. Despite the prevalence of HCC in China, East‐South Asia, some of the Pacific islands and the sub‐Saharan region are predominantly affected by HBV infection. In contrast, HCV is the major risk factor in populations located in Korea, Italy, Taiwan, and Western countries [7, 8]. HBV‐induced HCC incidence is decreasing due to effective anti‐viral therapeutic modalities. However, HCV‐HCC cases are continuously increasing worldwide [5]. Alcoholic liver diseases (ALD), such as alcoholic steatohepatitis (ASH) and alcoholic hepatitis (AH), are another major HCC risk [9]. ALD is a silent liver disease, with an absence of characteristic symptoms in its early phase, which results in lack of proper treatment and delay in diagnosis. Thus, ALD can progress to steatohepatitis and cirrhosis, the major causes of HCC. Indeed, ALD‐induced HCC accounts for 33% and 18% of total HCC cases in men and women, respectively [10], and ALD‐related cirrhosis is responsible for 45% of all liver cirrhosis‐induced deaths in 2010 [11]. Moreover, long‐lasting inflammation due to synergistic effects between multiple HCC etiologies, such as viral infection, heavy alcohol consumption, and metabolic stress, leads to liver damage and worse prognosis of HCC. Such topics have been intensively reviewed in previous articles [8, 10, 12, 13, 14]. Therefore, we will focus on the role of metabolic dysfunction‐associated fatty liver disease (MAFLD) in cirrhosis and HCC progression in this review (Fig. 1).

## 3 Obesity and metabolic syndromes

Obesity is a pandemic, metabolic disorder, affecting nearly a third of the worldwide population [15, 16, 17]. The prevalence of obesity has greatly increased in past decades in the US and worldwide due to rapidly developing food industries serving convenient, caloric‐dense foods, which induce longstanding positive energy balance in the body [18, 19]. Moreover, remarkable improvement of technical devices facilitating a sedentary lifestyle accelerates the formation of an obesogenic environment. Obesity increases the incidence of other metabolic diseases, including type 2 diabetes (T2D) and MAFLD, and the healthcare cost allocated to treat obesity and its related comorbidities is projected to exceed $150 billion in the US [20]. Moreover, obesity acts as an independent risk factor of HCC, with a 2‐fold rise of cancer incidence in obese patients [21] and body mass index (BMI) over 40 is proportionally correlated with an elevated risk of death in patients with HCC [22]. Meta analysis found that obesity accompanies metabolic syndrome (MetS), such as hypertension, hyperinsulinemia, and hyperglycemia [15], of which prevalence raises HCC incidence by 1.8 fold among obese individuals [23, 24, 25]. Among other MetS, hyperglycemia and obesity significantly increased HCC incidence, while hypertension and hypercholesterolemia did not show meaningful correlation [26, 27], emphasizing the key role of glucose in regulation of hepatocyte homeostasis. Hyperglycemia, a major symptom of patients with T2D, is a clinical sign of perturbed glucose homeostasis and is mostly co‐incident with hyperinsulinemia due to excessive fatness of patients with obesity and T2D [28]. In T2D patients, the risk of cirrhosis is significantly elevated, and there is a 2‐fold increase in HCC [29, 30]. When T2D co‐exists with obesity, it synergistically increases the risk of HCC [26, 30]. The underpinning mechanism that links MetS and HCC can be chronic, low‐grade inflammation, arising from adipocytes laden with excessive fats and accompanied with perturbation in insulin signaling, metabolic regulation, and organellar functions [31, 32].

## 4 Metabolic dysfunction‐associated steatohepatitis

Metabolic dysfunction‐associated steatohepatitis (MASH) is a heterogeneous liver disease, of which incidence was led by multiple etiologies, including obesity, MetS, and genetic risk [33]. Thus, MASH is a complex liver disease, and its worsening leads to cirrhosis, a major cause of HCC [34, 35]. Simple steatosis that includes fat accumulation with no major inflammation progresses to MASH, characterized by ectopic fat accumulation in more than 5% of hepatocytes along with extensive inflammation [34]. Less than 10% of simple steatosis patients develop MASH, suggesting that molecular mechanisms underlying MASH progression affect a minor portion of fatty liver patients but determine MASH initiation. Cumulative studies indicate that MASH is developed by the dysregulation of multiple key pathways that result in the formation of inflammatory foci, many of which can lead to compensatory cirrhosis [36]. The serial liver biopsies from 103 patients found that 37% of MAFLD patients progress to fibrosis [37]. A follow‐up study of 17 years indicates that up to 12.5% of MASH patients progress to cirrhosis whereas less than 1% of simple steatosis cases develop cirrhosis [35]. A retrospective study conducted between 1975 through 2005 at medical centers in the US, Europe, and Thailand found that 33% of MAFLD patients underwent liver transplantation or died and their liver biopsies indicate these morbidity and mortality closely associated with liver fibrosis [38]. After stratified confounders, age, sex, BMI, and MetS, diagnosis of MASH is the best independent association with liver‐related mortality [36], and patients whose fibrosis score is more than three out of four, display increased HCC incidence and mortality [39]. However, due to a lack of surveillance program applicable to asymptomatic MASH patients, MASH patients were diagnosed at the late stage in which liver failure is irreversible and often required liver resection and transplantation.

Nonetheless, a number of MASH patients develop HCC without cirrhosis. Approximately 15% of HCC cases have been developed in the absence of clinically noticeable cirrhosis [40]. The molecular mechanisms distinguishing HCC in non‐cirrhotic MASH from cirrhotic MASH liver have not been elucidated yet but it is found that non‐cirrhotic HCC patients were older, severely obese, and associated with co‐existence of multiple metabolic complications [40, 41].

Another type of MASH is the MASH patients whose BMI is less than 22 in Asia or less than 25 in Western countries, called MAFLD in a lean individual. The prevalence of MAFLD in lean BMI varies across the world, with a higher incidence in Asia and other countries such as Austria, Mexico, and Sweden but less frequent in Malaysia and Pakistan [42]. Although lean individuals with MAFLD may look healthy, they display similar degree of dyslipidemia, insulin resistance, and hyperglycemia and carry single nucleotide variant (SNV) of the genes such as *PNPLA3*, *CEPT*, and *PEMT*, all of which are important for regulation of hepatic lipid and choline metabolism [43].

Collectively, it indicates that molecular mechanisms underlying MASH‐derived HCC progression are various and complicated [44].

### 4.1 Comorbidities of MASH

T2D is an important independent risk factor of MASH, increasing MASH incidence two‐fold, and the prevalence of MASH among patients with T2D is 37% [45, 46, 47]. Moreover, appearance of advanced fibrosis was two times higher in obese patients with T2D, compared to those without T2D [48], and a cross‐sectional study conducted in Malaysia found that among T2D patients, prevalence of MASH is 81% and fibrosis is 31% [45]. These studies indicate that MASH and T2D share important pathologic pathways and the presence of T2D stimulates the adverse clinical outcome of MASH.

Of interest, MAFLD liver shares similar pathologic spectra with steatotic ALD. Liver biopsies from ALD were SA‐β‐gal staining‐positive and showed profound p21 activation, suggesting that alcohol‐induced oxidative stress accounts for senescence in alcoholic steatohepatitis [49, 50]. Likewise, oxidative stress, p53 and p21, were significantly upregulated in MAFLD livers compared to simple steatosis, while cyclin A was markedly suppressed [51, 52]. ER stress serves as a key second hit of MASH progression. Upon lipotoxicity, ER stress upregulates lipid synthesis via activation of sterol regulated element binding protein (SREBP), a master regulator of hepatic lipogenesis [53, 54]. While alcohol intake generated homocysteine that results in hepatic ER stress, excessive fructose consumption and lipotoxicity gave rise to ER stress in the MAFLD liver [55, 56]. Moreover, we identified that caspase2 (Casp2) promoted proteolytic activation of site‐1 protease (S1P), resulting in SREBP activation in response to ER stress [57, 58]. Importantly, Casp2, S1P, SREBPs are highly upregulated in both MASH and ALD livers [59]. Collectively, oxidative stress, senescence, and ER stress are key shared pathologies between ALD and MAFLD progression. Thus, mice fed a high‐caloric diet with alcoholic drinks have been used to study the molecular mechanisms underlying steatotic ALD progression [59].

## 5 Diagnosis of MAFLD in clinic and preclinical animal models

According to guidelines released from the American Association for the Study of Liver Disease (AASLD) and the National Institute of Diabetes and Digestive and Kidney Diseases (NIDDK), MAFLD diagnosis requires histological evidence of hepatic steatosis, detected by either magnetic resonance imaging or histology assessment, and co‐existing etiologies for chronic liver diseases, such as significant alcohol consumption, chronic viral hepatitis, drug‐induced liver damage and Wilson's diseases, must be excluded [60, 61]. Liver biopsy is the most reliable method of MAFLD diagnosis, giving the scores indicating the severity of disease, called NAFLD activating score (NAS) [62]. Indeed, the NAS score is used as an indicator of disease prognosis. However, a biopsy test has a limitation due to its costly procedure, sampling error, and post‐surgery complications. Therefore, a non‐invasive, reliable assessment method is strongly recommended [60, 61, 62]. The presence of MetS has been considered a strong non‐invasive predictor of MAFLD as insulin resistance and dyslipidemia are positively correlated with the severity of liver fibrosis. A cross‐sectional case study performed with 10 724 Korean individuals found that the hepatic steatosis index (HIS), which includes BMI, the presence of diabetes mellitus (DM), waist circumference (WC), plasma liver enzyme, serves as a risk indicator of MASH [63]. Moreover, the hepatic liver index (HLI) consists of BMI, WC, plasma triglycerides (TG), and gamma‐glutamyltransferase (GGT) and has also been suggested as a predictive factor of MAFLD and validated against ultrasonography [64]. The NAFLD liver fat score (NLFS) was used to evaluate the measurement of liver fat content from 470 patients and suggested that the presence of MetS, an increased fasting serum insulin and plasma glucose, and a decreased aspartate aminotransferase/alanine aminotransferase ratio can be a strong MASH predictor [65]. However, these non‐invasive blood markers provide modest efficacy in detecting MAFLD and still need imaging tools for accurate evaluation.

## 6 Genetic variations linked MASH‐driven HCC

The landscape of HCC genetic alterations has been mapped by high‐throughput analyses of a large number of samples and has identified the various mutational signatures and molecular pathways in HCC [66]. Research study using data from the cancer genome atlas (TCGA) identified that *TERT*, cell cycle regulator *TP53*, *AXIN1*, and *RB1* genes, WNT pathway oncogene *CTNNB1*, and *ARID1A*, *ARID2*, and *BAP1* which are chromatin remodeling players were significantly mutated in HCC [67]. With whole exome sequencing using MASH‐driven HCC and other etiologies‐induced HCC, Pinyol et al. [68], found that the most frequent HCC drivers in MASH patients were *TERT* promoter mutations, *CTNNB1* and *TP53* SNVs, and a TGF family activin receptor, *ACVR2A*, mutation. Importantly, MASH‐driven HCC showed a higher mutation rate of *ACVR2A* but a lower rate of *TP53* SNV compared to alcoholic and viral HCC [68]. Mutation signature analysis revealed that a mutational characteristic that appeared exclusively in MASH‐driven HCC is a higher incidence of C > T and C > A transitions [68]. In line with this, *TERT* promoter mutations, affecting nearly 44% of MASH‐HCC patients, are C228T and C250T transitions [67]. In addition, the *PNPLA3* rs738409 variant that substitutes isoleucine at position 148 to methionine (I148M) is frequently found in patients with MASH and is associated with a high risk of cirrhosis‐associated HCC onset [69, 70, 71]. Other SNVs of genes regulating iron metabolism [72], copper accumulation [73], DNA damage repair program [74], cell cycle regulation [75, 76], and inflammatory cytokines [76, 77] are also found in MASH‐driven HCC biopsies. In addition, whole exome sequencing and mutational signatures analyses revealed that non‐cirrhotic HCC exhibited significantly higher mutation rate in given HCC drivers compared to cirrhotic MASH‐HCC [68].

To delineate the malignant role of mutation in the process of MASH to HCC transition, tiny liver pieces were isolated from normal liver and cirrhotic nodules separated by fibrous septae which were subjected to mutation analysis. Normal hepatocytes exhibited polyclonal status, with limited genetic relatedness whereas liver micro‐biopsies obtained from cirrhotic nodules presented monoclonality with various mutations, and mutation patterns from each cirrhotic nodule were not shared if microsections were separated by fibrous septa. This indicated that cirrhosis is the early event that may be initiated with liver injury simultaneously and mutations evolve independently in many times in regenerative cirrhotic nodules of same patient liver [78]. Of note, *FOXO1*, *CIDEB*, *GPAM*, and *AVCR2A* SNVs were recurrent in MASH liver but not in healthy controls and importantly, these mutations were not found in HCC, suggesting that SNVs in MAFLD hepatocyte likely function against tumorigenesis [78, 79]. Later, Wang et al. [80], showed that these non‐cancerous mutants protected hepatocytes from lipotoxicity, thereby promoting survival of injured hepatocytes. Collectively, these studies demonstrated that these non‐cancerous SNVs are convergent evolutional outcomes that are positively selected for hepatocyte fitness and survival.

## 7 Adipocyte‐driven liver aggravation

The presence of excessive fat often results in blunted insulin sensitivity and increases lipid mobilization [81, 82]. Insulin resistance (IR), a metabolic syndrome found in patients with obesity, is suggested to be a major cause of MAFLD due to its regulation of multiple pathways required for maintenance of hepatocyte proliferation, growth, and survival. Insulin‐bound insulin receptor activates its tyrosine kinase domain that in turn catalyzes phosphorylation of insulin receptor substrate 1 or 2 (IRS1/2). This IRS1/2 activation transduces the signal to multiple pathways regulating phosphatidyl inositol 3 kinase, mitogen activating protein kinase (MAPK), and mTORC1 [81]. As such, mutations of insulin‐regulated pathways were frequently found in neoplastic cells [83], and expression of insulin receptor is upregulated in HCC livers [84]. However, the role of obesity‐induced IR in MASH and cirrhosis progression remains unclear. It has been suggested that selective IR, which selectively loses its suppressive effect on hepatic gluconeogenesis while retaining its promotive ability to hepatic lipogenesis, is the underlying mechanism of hyperglycemia and aggravation of lipotoxicity [85, 86]. However, in response to glucose intake, obese patients with MAFLD failed to carry out lipogenesis while hepatic lipid synthesis in obese patients with a normal liver could be carried out normally [87]. Morbidly obese patients showed high circulating levels of leptin, a pro‐fibrogenic adipokine, but lower levels of adiponectin, an anti‐fibrogenic adipokine [88]. In line with this, *ob/ob* mice lacking leptin action are protected from liver fibrosis [89]. These studies suggest that IR may affect MASH‐driven HCC progression in an indirect manner.

## 8 Fat mobilization and lipotoxicity

The major site for deposition of dietary fats is known to be visceral adipose tissue (VAT), which has a great potential for hyperplasia and fat expansion according to positive energy balance [90, 91]. Enlarged VAT undergoes chronic inflammation, resulting from adipose tissue macrophage (ATM) activation, and leads to IR [31, 81], the major cause of uncontrolled fat mobilization. Lipolysis is a complicated molecular process that requires coordinated activation of multiple players, such as adipocyte triglyceride lipase (ATGL), hormone‐sensitive lipase (HSL), and monoglyceride lipase (MGL) [92]. ATGL and HSL are rate limiting enzymes and the activation of lipolysis is tightly regulated by hormonal reactions. More specifically, Catecholamines regulate ATGL and HSL through G‐coupled protein receptor‐mediated activation of cyclic AMP‐protein kinase A (PKA), resulting in the release of free fatty acid (FFA) and glycerol from TG. Additionally, Insulin suppresses ATGL through the activation of inhibitory Gα‐protein‐coupled receptor [92]. Thus, balance toward blunted insulin sensitivity allows the increase in lipolysis and releases free fatty acids (FFAs) into circulation, where elevated FFAs exert lipotoxic effects in multiple tissues [32].

Saturated fatty acids (SFAs) induce liver damage by altering membrane fluidity and composition [93], and by triggering proinflammatory macrophage activation [90]. This results in activation of proinflammatory signaling responses such as c‐Jun NH2‐terminal kinase (JNK)‐activator protein 1 (AP‐1) and IκB kinase (IKK)‐NF‐κB [94]. Inflammatory macrophages produce tumor necrosis factor (TNF), a multifunctional cytokine that plays an important role in obesity‐associated chronic inflammation [95]. The two forms of TNF are a 26‐kDa transmembrane trimer (mbTNF) and a 17‐kDa soluble trimer (sTNF), both of which bind to either TNF receptor 1 (TNFR1) or TNFR2, a cell‐surface homotrimeric receptor [95]. TNF‐bound TNFR promotes the formation of an intracellular signaling complex, called complex I, composed of TRADD, TRAF2, cIAP, and ubiquitinated receptor‐interacting protein kinase 1 (RIPK1). When RIPK1 is deubiquitinated, complex I converts to complex II and initiates the cell death program [96], a process that is suppressed by NF‐κB activation [97]. Cumulative studies demonstrated that TNF reprograms metabolic pathways. Namely, TNF deactivates insulin signaling by phosphorylation of an inhibitory serine residue on the insulin receptor [98], enhances lipolysis in adipocytes [99], and delays glucose clearance [100], all of which is regulated by TNF downstream targets, NF‐κB [101, 102], JNK [103], and mitogen‐activated protein kinase kinase (MEK1/2) [104]. TNF also induces accumulation of toxic lipid species in adipose tissue, such as ceramides that upregulated in circulation of obese subjects [105, 106], and TNF expression is elevated in obese individuals with MASH compared to non‐MASH group [106]. Moreover, TNF expression is correlated with the severity of MASH, presenting substantial TNF elevation in those with advanced fibrosis [107]. Genetic alteration of TNF associates with MAFLD, showing that SNVs of the *TNF* gene were frequently found in metastatic colorectal cancer patients whose livers are MAFLD [108]. TNF is also upregulated in multiple mouse models that are accompanied with obesity and metabolic complications [109, 110, 111]. Accordingly, inhibition of TNF protects from metabolic syndromes [112, 113, 114, 115], MASH and HCC development [110, 111, 116]. Mice lacking TNF or when administered with a TNF antibody were protected from IR and had reduced serum FFAs [95, 112, 114, 115]. TNFR1 knock out (KO) ameliorated HCC progression in obese mice [110] and prevented adipocyte FFA release [116], resulting in reduction in TLR4 signaling [94], oxidative stress [117], and apoptosis [118]. However, the acting mechanism underlying TNF‐regulated metabolic syndromes and MASH‐driven HCC progression in humans remains unclear. Despite alterations in metabolic parameters in many mouse models, treatment with a TNF inhibitor did not show improvement in insulin sensitivity in obese patients [113, 119, 120]. However, when patients with rheumatoid arthritis, whose mortality is associated with IR‐associated metabolic disorder, were treated with a TNF inhibitor, it significantly improved their insulin response, implying that TNF action is more complex in clinical contexts.

### 8.1 Hepatic ER stress and cell death

The ER is the largest membranous organelle that controls secretory proteins whose destination is the extracellular milieu [54]. In the lumen of the ER, secretory proteins undergo quality control that ensures their proper folding. When lipotoxicity initiatives, ER client proteins are directed to a salvage pathway operated by the unfolded protein response (UPR) [54], a defense system that addresses ER stress. UPR is comprised of three transmembrane receptors, inositol‐requiring protein1 (IRE1), PKR‐like ER kinase (PERK), and activating transcription factor 6 (ATF6) [121, 122, 123]. Once activated by sensing the degree of unfolded client proteins [124], UPR signaling pathway mitigates ER stress by upregulation of ER chaperons, translational control of newly synthesized proteins, and expansion of the ER membrane.

IRE1, an evolutionarily conserved UPR stress sensor, possess a cytosolic serine/threonine kinase and an endoribonuclease (RNase) domain [125]. By increasing unfolded client proteins in the ER lumen, IRE1 is oligomerized and activates its RNase activity that cleaves the phosphodiester bonds of two native RNAs of XBP1 and removes intervening introns [126]. This results in the formation of an activated XBP1 basic leucine zipper (bZIP) transcription factor that induces the expression of genes encoding ER‐associated protein degradation (ERAD) machinery and ATF6 [122]. Unlike the IRE1 tetramer required for XBP1 splicing, the IRE1 dimer processes micro RNAs (miRNA) [127]. ATF6 is a type II transmembrane bZIP protein, located at the ER membrane as a precursor [128] and exists in two isoforms, ATF6α and β, both of which are expressed ubiquitously [129]. Membrane‐bound precursor ATF6(p) undergoes ER stress‐induced proteolytic cleavage by S1P and site‐2 protease (S2P) to release a N‐terminal transcription factor [130] that binds to ER stress response element (ERSE), resulting in the expression of a number of ER chaperons such as Grp78, Grp94 and calnexin [131]. It is found that ATF6 forms a dimer with XBP1 and stimulates expression of ER stress responders, implying that UPR arms are interconnected and respond to ER stress cooperatively [123, 132]. The third ER stress receptor is PERK, which plays a role in translational control in response to ER stress. PERK is an ER‐resident type 1 transmembrane receptor, the N‐terminal domain of which binds to GRP78/Bip, and its cytosolic C‐terminal end retains a kinase domain that phosphorylates eukaryotic translation initiation factor 2α (eIF2α) [132]. PERK phosphorylates serine at position 51 of eIF2α, which results in inhibition of guanine nucleotide exchange factor for eIF2 and reduction of translational rate of general proteins [133]. Along with IRE1‐regulated ERAD [134], PERK‐induced eif2α phosphorylation leads to a decline in client protein load in the ER [132, 133]. On the contrary, phosphorylated eIF2α selectively promotes the translation and expression of activating transcription factor 4 (ATF4) by allowing ribosomes to bind to upstream open reading frame 1 (uORF1), generating full‐length ATF4 [135, 136]. In turn, ATF4 suppresses eIF2α through regulation of GADD34, a phosphatase that dephosphorylates eIF2α, thereby allowing cells to re‐initiate translation [137]. Moreover, ATF4 assists the process of protein synthesis by increasing amino acid transportation [138]. However, when ER stress is not resolved, C/EBP homologous protein (CHOP) induces cell death [139].

Of note, UPR is upregulated in MASH compared to simple fatty livers [140, 141], and stimulated by refined sugar, of which consumption is proportionally increased with MAFLD incidence [56, 58, 142]. Fructose, one of refined sugars that is a frequently used dietary sweetener, presented in fruits and honey, and its usage in a form of high fructose corn syrup was proportionally increased with the incidence of metabolic disease [56, 143, 144, 145]. Intake of fructose increases plasma lipids, hepatic *de novo* lipogenesis (DNL), fasting glucose output, and decreases insulin sensitivity in individuals given a fructose beverage compared to those consuming a glucose drink [146, 147], indicating the potential link between dietary fructose and hepatic metabolic perturbation. Cumulative studies suggested that fructose stimulates hepatic DNL independent of insulin action. Although fructose stimulates pancreatic insulin secretion marginally, fructose is fully capable of hepatic lipid synthesis in obese individuals with MAFLD [87, 148]. As increased DNL is a unique feature underlying MAFLD development, accounting for 24% of hepatic fat source [57, 149, 150], elucidating the molecular mechanism responsible for fructose‐induced hepatic DNL is important for MASH treatment. We found that ER stress activates Casp2, the most conserved cysteine protease and Casp2 stimulates hepatic SREBP and DNL through S1P proteolytic activation [57, 58]. Moreover, we found that Casp2‐mediated hepatic SREBP activation relies on intake of SFA and fructose [58]. In line with this, Casp2 is highly upregulated in patients with MAFLD compared to those with simple fatty livers [57, 59].

Lipotoxicity triggers a cell death program in the MASH livers and hepatocyte death is one of the pathologic features distinguishing MASH liver from simple steatosis [151, 152, 153]. Prolonged exposure of FFAs stimulated CHOP‐mediated cell death through upregulation of p53 upregulated modulator of apoptosis (PUMA) in Huh‐7 cells [154]. CHOP also induced expression of death receptor 5 (DR5) followed by caspase 8 activation, resulting in apoptosis [155, 156]. RIP3 is a necrosome component that activates a downstream necrosis effector, MLKL, and is upregulated in MASH livers [153]. Although the pathological consequence of MLKL‐dependent hepatocyte death in MASH progression is not entirely clear, recent studies suggested that hepatic ferroptosis plays an important role in initiating inflammation of the liver and ATF4 protects fatty hepatocytes from ferroptosis in preclinical model [157, 158]. Another type of programmed cell death is pyroptosis, which requires activation of the inflammasome complex, components of which include NLR family, pyrin domain containing 3 (NLRP3), apoptosis‐associated speck‐like protein containing a CARD (ASC) and procaspase‐1 [159]. When the inflammasome was activated by pathogen‐associated molecular pattern, activated caspase 1 induced proteolytic activation of IL‐1 and IL‐18, resulting in pyroptotic cell death. Importantly, active caspase‐1 proportionally increased the severity of MASH [159, 160, 161]. The mechanisms underlying MASH‐related cell deaths and their impacts on MASH progression have been described in previous review articles [155, 162].

## 9 Protective role of hepatic TG

Lipid accumulation is the earliest hepatic manifestation of MAFLD [53], and hepatic TG is thought to lead to liver aggravation and MASH progression. However, the relationship of liver steatosis, IR, and liver damage is more complex than expected. Saturated fatty acids (SFAs) cause ER stress and induce accumulation of diacylglycerol (DAG) in the livers of MAFLD patients [32, 163, 164, 165, 166, 167]. DAGs are esters of trihydric alcohol in which two hydroxyl groups are esterified by fatty acids. By esterifying DAG, diacylglycerol‐O‐acyltransferase (DGAT) catalyzes the final step of TG biosynthesis and an elevated hepatic DGAT's activity has been implicated in IR, ER stress and liver damage [166]. To determine the relationship between liver damage and steatosis, Monetti et al. [168] overexpressed DGAT2 in the liver of mice (Liv‐DGAT2) and found that Liv‐DGAT2 mice increased hepatic TG, DAG and ceramide spontaneously but their livers showed normal glucose and insulin tolerance and did not exhibit elevated inflammation, a prerequisite of MASH and HCC progression. Moreover, Yamaguchi et al. [169] inhibited DGAT2 by administration of anti‐sense oligo (ASO) to methionine and choline‐deficient diet‐fed *db/db* mice. DGAT2 inhibition successfully increased liver FFA level more than two‐fold and liver steatosis was extensively reduced in the DGAT2 ASO‐treated group. However, the DGAT2 ASO‐treated group increased liver damage, mitochondrial FFA, elevated oxidative stress, liver damage and expression of fibrogenic enzymes, all of which promotes cirrhosis progression. These studies demonstrated that neutral fat accumulation protects the liver from FFA‐mediated lipotoxicity. In line with this, FFA administration led to ER stress, accumulated lipids, and cell death in hepatocytes [167]. Moreover, incomplete TG formation can also lead to upregulated DAG, a second messenger, that activates protein kinase C (PKC), JNK and NF‐κB, all of which promote IR [170]. In support of these findings, we [58] and the Nakagawa group [171] found that ablation of SREBP cleavage activating protein (SCAP), a chaperon required for SREBP activation and stabilization [172], blocked SREBP1/2 activation and fat and cholesterol (Chol) biosynthesis but greatly increased liver damage and inflammation. These studies indicate that more subtle and sophisticated approaches are required to understand the nature of stress‐regulated hepatic lipid metabolism.

## 10 Mouse models of MASH‐driven HCC

### 10.1 Diet‐induced MASH‐driven HCC rodent models

MASH is a metabolic liver disorder, frequently associated with a prolonged intake of high‐caloric diets [142, 173]. Therefore, it is not surprising that MASH‐driven HCC incidence is caused by lifestyle‐related risk factors [173]. Several studies indicate that adherence to intake of processed meat and refined sugars promotes HCC incidence whereas a Mediterranean dietary pattern and an urban prudent eating pattern are protective [174]. A high‐fat diet (HFD), providing 60% of calories from lard, is the most common rodent diet that induces obesity and MAFLD. By HFD intake, small animals gradually increase body weight, predominantly through adipocyte hyperplasia and hypertrophy that perturb the systemic insulin response, resulting in ectopic fat accumulation and development of steatosis [175, 176]. Several studies have shown that HFD‐induced obesity provokes insulin resistance but seldomly progresses to MASH and HCC in the absence of extra manifestations required for inflammation and hepatocyte damage [175, 176]. Thus, introduction of genetic mutation and cell death by the chemical carcinogen, diethylnitrosamine (DEN), can lead to tumorigenesis in mice fed a HFD, and inhibition of inflammation reduces the degree of obesity and incidence of HCC [111]. The *ob/ob* mice, which have defective leptin signaling, become obese when fed normal chow and develop fatty liver without fibrosis, attesting to the requirement of liver damage for MASH progression from simple steatosis. When injected with DEN, *ob/ob* mice developed HCC [111], in line with the notion that obesity acts as a tumor environment where malignant hepatocytes are fostered, but is not a carcinogen *per se*. Therefore, HFD is often combined with other dietary components and tested for MASH and HCC development in preclinical models. A high fat and high carbohydrate (HFHC) diet has also been developed. This diet consists of saturated fat and carbohydrate, supplemented with high fructose and glucose water. HFHC diet‐fed C57BL/6 mice became obese with extensive subcutaneous and visceral adiposity, exhibited metabolic syndromes like insulin resistance, and developed MASH‐like phenotypes presenting macrosteatosis with elevated liver TG and FFA content, inflammatory responses, ballooning degeneration and mild fibrosis. When fed over a longer period of time, HFHC diet stimulates hepatocyte death and proliferation, which potentiates hepatic neoplastic transition [177, 178]. However, similar to the HFD effect, a fructose diet induces liver steatosis but minimal inflammation and damage [58]. Knock‐out of ketohexokinase (KHK), a rate limiting enzyme of fructolysis, ameliorates MASH progression upon HFHC intake, supporting the necessary role of fructose in MASH development [179]. Chol is a lipid metabolite required for membrane generation and signal transduction. Hence, dysregulation of hepatic Chol metabolism is implicated in oxidative stress, cell death, and stellate cell activation [142]. In line with the protective role of TG, it is found that high‐fat diet protects from oxidative stress while Chol damages the liver and increases inflammation [142, 180, 181]. A Western diet (WD), similar to the fast food diet consumed in Western countries, is composed of saturated fats, carbohydrates, and Chol ranging from 0.1% to 2% of total weight [182]. When C57BL/6 mice were fed a WD for 6 months, mice developed obesity with dyslipidemia and elevated plasma liver enzymes and exhibited MASH presenting two‐fold increased liver macro‐ and micro‐steatosis, extensive inflammatory infiltration, hepatocyte ballooning, and stellate cell activation compared to the HFD‐fed controls [182, 183]. By giving WD supplemented with fructose and glucose water (WD‐SW) to isogenic B6/129 mice generated by a cross between C57BL/6J and 129S1/SvImj strains for a year or more, called a DIAMOND model, mice accelerated HCC development with the appearances of nodularity in some of livers and multiple foci of tumors with hemorrhage within some of larger tumors. Mice presented grade 3 liver steatosis at 8 weeks and gradually decreased by 52 weeks at which ballooning and inflammation were prominent. In addition, mice exhibited a fibrosis pattern resembling that seen in human MASH [184]. Gene Ontology (GO) annotation analysis of human simple fatty liver, early MASH, and advanced MASH livers identified that gene sets including ribosome stress, senescence and metabolic pathways were enriched in early MASH but become less enriched so as ballooning, fibrosis, focal adhesion, and extracellular (ECM)‐receptor interaction were progressed in advanced MASH [185, 186]. WD‐SW fed B6/129 mice showed that apoptosis, cell proliferation, and inflammation pathways were enriched in livers of 8‐week‐fed mice and gene sets regulating oxidative stress, innate immune system and inflammatory pathway were markedly elevated in livers of 52 weeks‐fed mice [184, 187]. Mice with the *Alms1* mutation (*foz/foz* mice) given WD developed MASH with extensive steatosis, inflammation and grade 2 to 3 fibrosis at their 12 weeks of feeding and developed visible tumors and cirrhosis in the non‐tumor area at 24 weeks [188]. Transcriptome analysis revealed that 254 genes, including key genes such as *COL1A1*, *LGALS3*, *SPP1*, and *Trem2*, were dysregulated in the livers of both WD‐fed *foz/foz* mice and human MASH patients and found that these genes regulate cell to cell interaction and immune responses [188]. Moreover, gene expression analysis identified that among diet‐induced mouse MASH‐driven HCC models, livers of WD‐SW fed mice presented gene expression patterns resemble those of MASH‐driven HCC patients [68]. Clapper et al., replaced a portion of lard in HFD with trans fats originated from hydrogenated vegetable oil and increased Chol component by 2% of total weight, naming this the Amylin liver NASH (AMLN) diet. After consumption of an AMLN diet for 30 weeks, mice presented marked increases in several MASH markers similar to those found in human MASH, such as insulin resistance, macro‐ and micro‐steatosis, inflammation, fibrosis, ballooned hepatocytes, and collagen deposition [189, 190]. Bulk quantitative transcriptome and single cell analyses in AMLN‐fed mice revealed that while hepatocytes were enriched in gene sets regulating fat metabolism, glucose/fructose and mannose metabolism, the hepatic stellate cell (HSC) subpopulation was enriched in gene sets that stimulate HSC activation and trans‐differentiation [191, 192]. The AMLN diet was combined with limited physical activity to mimic the sedentary lifestyle of modern people, named American lifestyle‐induced obesity syndrome (ALIOS) diet [193]. When ALIOS‐fed mice were given a high fructose corn syrup (HFCS) equivalent, they gained 10% more body weight (BW) than when fed a trans fats diet with no HFCS consumption and 42% more than the control group, and displayed increased liver TG and plasma Chol by 16 weeks [193]. When B6/129 mice were fed an ALIOS diet for a year or more, 50% of mice developed neoplastic lesions, with presentation of HCC markers, Sox9 and β‐catenin [194]. ALIOS‐fed mice also presented with liver inflammation, cirrhosis, hepatomegaly, and glucose tolerance which are also found in MASH‐driven HCC patients. Importantly, steatosis and lipogenic enzymes in ALIOS‐fed mouse livers reached peak expression at 6 months of feeding, after which their expression reduced as disease worsened [194], similar to burned‐out MASH found in patients with end‐stage liver disease [195, 196]. However, the ALIOS diet did not increase plasma TG and hepatocyte ballooning, representative anthropometric measurements of MASH in patients [194]. Transcriptome analysis of the livers of ALIOS diet‐fed mice found that among differentially expressed genes, 22.5% of genes were shared between livers from human MASH patients and ALIOS‐fed mice [197].

### 10.2 Choline‐deficient diet

Methionine and choline‐deficient diet is often used to induce MASH within a short period of time in mice or rats, as it causes extensive liver steatosis, inflammation, and severe fibrosis. However, a serious shortcoming of this diet is a substantial reduction in BW and liver weight, and lack of MetS, which is inconsistent with the anthropometric characteristics of MASH patients [198, 199, 200]. Choline‐deficient and L‐amino acid defined diet (CDAA) is also based on a lack of dietary choline but substitutes proteins with an equivalent mixture L‐amino acids, resulting in prevention of drastic weight loss and stimulation of liver steatosis by inhibiting VLDL secretion [201]. When fed to Wister rats, liver steatosis was seen after 1 week of feeding and remained unchanged throughout the feeding period. Inflammation score peaked at 12 weeks and gradually decreased afterwards, while fibrosis began around 12 weeks and kept increasing until the 24 weeks [202, 203]. Similarly, CDAA fed C57BL/6 mice developed liver steatosis and fibrosis at their 22‐week of feeding [201]. However, a CDAA diet led to significant BW reduction in C57BL/6 mice and Wister rats compared to those fed HFD [201, 204]. Given that weight management is one of the best effective therapeutic approaches for patients with early MASH, a detailed investigation of molecular pathways underlying CDAA‐induced MASH‐driven HCC formation is warranted. When a CDAA diet was given along with a HFD (CD‐HFD), C57BL/6J mice developed MASH, presenting 3 grade of steatosis, 1.5 and 2 grade of inflammation and fibrosis score, respectively, and ballooned degeneration at 24 weeks and induced HCC at 60 weeks, indicating CDAA‐HFD induces MASH‐driven HCC in C57BL/6 mice [204, 205]. However, CDAA‐HFD diet‐induced MASH phenotypes did not include elevated blood TG, a representative feature of obesity‐associated MASH patients [201, 204].

### 10.3 Chemical and genetic mouse models

Streptozotocin (STZ) is a nitrosourea alkylating agent that selectively induces pancreatic β cell death. As a result, it causes insulin deficiency and hyperglycemia, characteristics of type 1 diabetes [206]. C57BL/6 mice were given low‐dose streptozotocin (STZ) 2 days after birth and were fed HFD from the 4th week for 8 to 20 weeks, which is called a STAM mouse model [207]. STAM model mice exhibited steatosis, inflammation, fibrosis, and ballooned hepatocyte degeneration at their 12 weeks and developed HCC by the week of 20th [207, 208, 209]. This has the advantage of causing mice to develop liver damage and neoplastic transition rapidly [207, 208], However, unlike MASH‐driven HCC patients whose anthropometric characteristics include being overweight and displaying hyperinsulinemia due to excessive positive energy balance [53], STZ‐injected mice exhibited low blood insulin and low body weight. Similarly, DEN, a DNA alkylating agent [210], is a potent carcinogen and when it was combined with HFD intake, mice developed HCC onset by 36 weeks but in the absence of MASH [111, 211]. By weekly dosing carbon tetrachloride (CCL4), livers of WD‐fed mice presented stage 3 fibrosis by 12 weeks and HCC by 24 weeks [212]. However, CCL4 treatment induces genotoxicity and oxidative DNA damage [213].

*Db/db* mice possess a mutation in leptin receptor [214] and are obese, IR, and diabetic [215]. Similar to *ob/ob* mice, *db/db* mice develop microvesicular steatosis but the degree of steatosis is more severe in *ob/ob* than in *db/db* mice and do not develop hepatic necroinflammation spontaneously [216]. Liver‐specific SREBP1 transgenic (nSrebf1c‐tg) mice showed marked liver steatosis and inflammation by 8 weeks, and by 20 weeks mice exhibited MASH markers, such as ballooning degeneration, Mallony Denk bodies, fibrosis, and such phenotypes were worsened as the feeding period was extended [217]. However, nSrebf1c‐tg mice lost mass of visceral fats [218]. PTEN, a negative regulator of phosphatidyl inositol 3‐kinase, was mutated in the liver of *Pten* ^Loxp/Loxp^;Alb‐CRE^+^ mice. Due to hyper‐phosphorylation of AKT and upregulated hepatic lipid synthesis, mice developed extensive liver steatosis, hepatomegaly, glucose intolerance, and hyperglycemia [219]. After 44 weeks, 47% of *Pten* ^Loxp/Loxp^;Alb‐CRE^+^ mice developed liver adenomas and 66% of mice had an onset of HCC by 74~78 weeks. However, *Pten* ^Loxp/Loxp^;Alb‐CRE^+^ mice showed hyper‐insulin sensitivity [220]. Acyl‐Coenzyme A (AOX) KO mice are defective in metabolizing long‐chain fatty acids, which accumulate in the liver and lead to fatty liver [221]. *AOX* ^−/−^ mice increased hepatic peroxisome proliferative receptorα and cytochrome p450, resulting in lipid accumulation by 4~5 months of age, but by 7~8 months, liver steatosis was resolved by compensatory increase in FA oxidation [221]. Methionine adenosyl transferase (MAT) catalyzes the formation of S‐adenosylmethionine (AdoMet). Deletion of the liver‐specific *MAT* gene encoding MAT1a increased plasma methionine level by 76% and by 3 months, mice showed increased liver weight and hepatocyte proliferation [222]. Although *MAT1a* ^−/−^ mice develop hepatic adenoma spontaneously by 18 months, their circulating insulin levels are normal and no other metabolic complications are observed [223]. *MUP‐uPA* mice induce the transgenic gene, urokinase plasminogen activator (*uPA*), which results in hepatocyte ER stress [53, 110], due to over production of a secretory uPA that accumulates in the ER [121]. Upon HFD feeding, *MUP‐uPA* mice developed MASH, with presentations of classical MASH markers, steatosis, inflammatory infiltration, Mallony Denk bodies [57], and mice given HFD for 40 weeks developed HCC [110, 224]. Of note, uPA expression found to induce genomic instability, upregulated hepatocyte proliferation, and inflammation in hepatocytes [225], implicating that several factors cooperate in MASH and HCC development in this model. Preclinical models described above are summarized in Table 1.

**Table 1:** MASH‐driven HCC animal models. √: Moderate expression (Score 1–2); √√: Strong expression (Score > 3); ALIOS: American lifestyle‐induced obesity syndrome (Harlan Teklad, Indianapolis, IN, USA, TD06303); AMLN: Amylin liver NASH model (Research Diet, New Brunswick, NJ, USA, D9100301); CDAA: Choline‐deficient, L‐AA defined diet (Dyets, Inc., Bethlehem, PA, USA, 518753); HFD: High‐fat diet (Bio‐Serv., Flemington, NJ, USA, S3282); HFHC: High fat, high carbohydrate diet supplemented with fructose water (Research Diet, D12492); MCD: Methionine and choline‐deficient diet (Harlan Teklad, TD06414); NA: Not assessed; WD: Western diet (Test Diet: AIN‐76, Western Diet: Harlan Teklad. TD88137); X: No expression; Y: HCC onset.

Diet‐induced MASH‐driven HCC rodent models									
Diet	Strain	Obesity	Insulin resistance	Steatosis	Inflammation	Fibrosis	Ballooning degeneration	HCC	References
HFD	C57BL/6J	√√	√√	√√	X	X	X	X	[175, 176]
HFHC	C57BL/6J	√√	√√	√√	√	√	√	Y	[177, 178]
WD	C57BL/6J	√√	√√	√	√√	√	√	NA	[182, 183]
WD	Foz/Foz	√√	√√	√√	√√	√√	√	Y	[188]
WD	C57BL/6 X 129Sl/Svlmj	√√	√√	√√	√	√√	√	Y	[184, 185]
AMLN	C57BL/6J	√√	√√	√√	√√	√	√	NA	[189, 190, 191, 192]
ALIOS	C57BL/6J	√√	√√	√	NA	NA	NA	NA	[193]
ALIOS	C57BL/6 X 129Sl/Svlmj	√√	NA	√√	√√	√√	X	Y	[194]
MCD	Wister Rat	X	X	√	√√	√√	X	X	[198, 199, 200]
CDAA	C57BL/6J	√	NA	√	√√	√	√	NA	[201]
CDAA	Wister Rat	√	NA	√√	√	√	NA	NA	[202, 203]
CDAA + HFD	C57BL/6J	√√	√√	√√	√	√	√	Y	[204, 205]

Chemical‐induced models									
Chemical + Diet	Strain	Obesity	Insulin Resistance	Steatosis	Inflammation	Fibrosis	Ballooning degeneration	HCC	References
STZ + HFD	C57BL/6J	X	X	√	√√	√√	X	Y	[207, 208, 209]
DEN + HFD	C57BL/6J	√√	√√	√√	√	√√	X	Y	[111, 211]
CCL4 + WD	C57BL/6J	X	X	√√	√	√	√	Y	[212]

Genetic models									
Gene Mutation	Strain	Obesity	Insulin Resistance	Steatosis	Inflammation	Fibrosis	Ballooning degeneration	HCC	References
Ob/ob	C57BL/6J	√√	√√	√√	X	X	X	X	[89]
Db/db	C57BL/ksj	√√	√√	√	X	X	X	X	[215, 216]
nSREBP‐1c	C57BL/6J	X	√√	√√	√	√	√	NA	[217, 218]
PTEN liver‐specific KO	C57BL/6J	X	√√	√√	NA	NA	NA	Y	[219, 220]
AOXKO	C57BL/6J	NA	NA	√√	NA	NA	NA	NA	[221]
MAT1KO	129Sl/Svlmj	NA	NA	√√	NA	NA	NA	Y	[222, 223]
MUP‐uPA + HFD	C57BL/6J	√√	√√	√√	√√	√√	√	Y	[53, 110, 224]

## 11 Conclusion and perspectives

There has been significant effort to develop MASH treatment that curtails HCC progression. Despite the growing incidence of MASH‐derived HCC, there is no difference in treatment of MASH‐derived HCC compared to other etiologies‐mediated HCC. The American Association of Clinical Endocrinology emphasized the weight management, dietary intervention, and management of MASH comorbidities. Thus, treatment of glucagon‐like receptor‐1 (GLP‐1) and PPAR agonist that helps obese patients lose weight and T2D patients manage hyperglycemia, respectively, has been widely applied to MASH patients. However, a treatment specific to MASH has not yet been discovered.

In comparison to non‐malignant hepatocytes, recent publications demonstrated that cirrhotic hepatocytes have increased mutational burden, presenting somatic mutations and chromosomal structural variants, as a result of chromothripsis, critical for malignant transition of normal hepatocytes [78]. Although these events are not yet included, future preclinical MASH models are engineered to have a key mutational signature and chromosomal variants, providing the molecular mechanisms underpinning how normal fatty hepatocytes attain malignant characteristics in MASH patients.

## Conflict of interest

The authors declare no conflict of interest.

## Author contributions

JYK conceived and designed the manuscript and MK helped editing the manuscript.
